# Prevalence and determinants of overweight and obesity in old age in Germany

**DOI:** 10.1186/s12877-015-0081-5

**Published:** 2015-07-14

**Authors:** André Hajek, Thomas Lehnert, Annette Ernst, Carolin Lange, Birgitt Wiese, Jana Prokein, Siegfried Weyerer, Jochen Werle, Michael Pentzek, Angela Fuchs, Tobias Luck, Horst Bickel, Edelgard Mösch, Kathrin Heser, Michael Wagner, Wolfgang Maier, Martin Scherer, Steffi G. Riedel-Heller, Hans-Helmut König, for the AgeCoDe Study Group

**Affiliations:** Department of Health Economics and Health Services Research, University Medical Center Hamburg-Eppendorf, Hamburg, Germany; Department of Primary Medical Care, Center for Psychosocial Medicine, University Medical Center, Hamburg-Eppendorf, Hamburg, Germany; Institute of General Practice, Hannover Medical School, Hannover, Germany; Central Institute of Mental Health, Medical Faculty Mannheim/Heidelberg University, Mannheim, Germany; Institute of General Practice, Medical Faculty Heinrich-Heine-University Düsseldorf, Düsseldorf, Germany; Institute of Social Medicine, Occupational Health and Public Health, University of Leipzig, Leipzig, Germany; Department of Psychiatry, Technical University of Munich, Munich, Germany; Department of Psychiatry, University of Bonn, Bonn, Germany; German Center for Neurodegenerative Diseases, Bonn, Germany

**Keywords:** Obesity, Overweight, Physical Activity, Elderly, Longitudinal

## Abstract

**Background:**

Mean body weight gradually increases with age. Yet, little data exists on the prevalence of excess weight in populations aged 80 years or older. Moreover, little is known about predictors of overweight and obesity in old age. Thus, the purpose of this study was: To present data on the prevalence of excess weight in old age in Germany, to investigate predictors of excess weight in a cross-sectional approach and to examine factors affecting excess weight in a longitudinal approach.

**Methods:**

Subjects consisted of 1,882 individuals aged 79 years or older. The course of excess weight was observed over 3 years. Excess weight was defined as follows: Overweight (25 kg/m^2^ ≤ BMI < 30 kg/m^2^) and obesity (BMI ≥ 30 kg/m^2^). We used fixed effects regressions to estimate effects of time dependent variables on BMI, and overweight or obesity, respectively.

**Results:**

The majority was overweight (40.0 %) or obese (13.7 %). Cross-sectional regressions revealed that BMI was positively associated with younger age, severe walking impairments and negatively associated with cognitive impairments. Excess weight was positively associated with younger age, elementary education, walking impairments and physical inactivity, while excess weight was negatively associated with cognitive impairment. Longitudinal regressions showed that age and severely impaired walking disabilities reduced BMI. The probability of transitions to excess weight decreased considerably with older age and occurrence of severe walking impairments (overweight).

**Conclusions:**

Marked differences between predictors in cross- and longitudinal setting exist, underlining the complex nature of excess weight in old age.

**Electronic supplementary material:**

The online version of this article (doi:10.1186/s12877-015-0081-5) contains supplementary material, which is available to authorized users.

## Background

Overweight and obesity are risk factors for numerous chronic diseases, like diabetes, hypertension, cardiovascular disease, osteoarthritis, and cancer [27, 32]. Health risks increase with excess weight, leading to substantial morbidity and disability, impaired quality of life, increased mortality, and perceived discrimination, which in turn is related to poor subjective health, lower life satisfaction, and feelings of loneliness [1, 3, 45]. The prevalence of excess weight has increased substantially in recent decades, with currently more than half (52.6 %) of the population in OECD countries being overweight or obese [33]. Consequently, excess weight is now considered one of the leading risk factors for non-communicable diseases in the developed world [16].

Mean body weight gradually increases with age [30, 31]. Based on the definition of obesity recommended by WHO (Body Mass Index, BMI ≥30 kg/m^2^), the prevalence of obesity in Germany in 2010 was highest in persons aged 70-79 years (M: 31.6 %, F: 41.6 %), which was markedly higher than in the general adult population (M: 23.3 %, F: 23.9 %) [31]. Yet, little data exists on the prevalence of excess weight in populations aged ≥ 80. While, e.g., a study from Spain reported a high prevalence of obesity in individuals aged ≥ 80 [20], no representative studies on the prevalence of obesity in old age exist for Germany where the population aged ≥ 80 is expected to triple until 2050 due to demographic change [5].

Moreover, little is known about predictors of overweight and obesity in old age. These might differ from younger population groups as in old age changes occur in body composition, height, food intake and energy expenditure [9, 18]. Old adults have more body fat which, in addition, is distributed differently. Likewise, a decrease in muscle mass and height is associated with ageing. Old adults tend to have a lower food intake and become less hungry. Furthermore, the degree of physical activities decreases in old age. Eventually, old adults frequently lose weight for reasons of frailty, morbidity and imminent death.

Internationally, existing data on excess weight in old age almost exclusively comes from cross-sectional studies. Hence, there is a lack of longitudinal studies that investigate factors affecting excess weight over time. Specifically, the impact of physical activity on excess weight has not yet been thoroughly researched. Only a few longitudinal studies [18, 39] investigated the influence of physical activity on body composition in individuals aged 65+. To our knowledge, no longitudinal study on physical activity affecting excess weight in individuals aged 80+ exists for Germany.

The purpose of this study was to present descriptive data on the prevalence of overweight and obesity in old age in Germany, to analyze associations of excess weight with socio-demographic variables and other possible predictors, in particular physical activity, in a cross-sectional approach, to investigate factors influencing excess weight using panel econometric techniques in a longitudinal approach.

## Methods

### Ethics statement

The ethics committees of the participating centers approved the study (reference numbers: 050/02 (University of Bonn), 2079 (Faculty of Medicine, University of Düsseldorf), 2817/2007 (Hamburg Medical Association), 309/2007 (Faculty of Medicine, University of Leipzig), 2007-253E-MA (Medical Ethics Commission II, University of Heidelberg at the University Medical Center of Mannheim), 713/02 (Faculty of Medicine, Technical University of Munich)). The study was conducted according to the principles expressed in the Declaration of Helsinki. All participants gave written informed consent prior to study entry.

### Sample

Data were collected as part of the German Study on Ageing, Cognition and Dementia in Primary Care Patients (AgeCoDe), which is a population based prospective cohort study. For AgeCoDe, general practitioners (GP) recruited n = 3,287 subjects aged ≥ 75 years at six cities in Germany (Bonn, Düsseldorf, Hamburg, Leipzig, Mannheim, Munich) in 2003 and 2004. Thereafter, trained research assistants interviewed participants at home every 1.5 years. Individuals were included if they met inclusion criteria at baseline: (a) age ≥ 75 years, (b) absence of dementia, and (c) at least one contact with the GP during the last 12 months. Consultations only by home visits, residence in a nursing home, insufficient German language skills, severe illness, deafness, blindness, and lack of ability to provide informed consent were exclusion criteria.

Height and weight was first assessed in the third follow-up wave (wave 3) in which n = 1,966 individuals participated. Hence, this study used data from follow-up wave 3 (4.5 years after baseline) up to follow-up wave 5 (7.5 years after baseline). Main reasons for lack of follow-up (follow up wave 1-3) data were refused participation (n = 712) and death (n = 508), while other reasons (n = 133) played a minor role. By the time of wave 4, 124 individuals died and 76 individuals refused participation (other reasons: n = 62). Between wave 4 and 5, 130 respondents died and 52 respondents were not interviewed at wave 5 for reasons of refusal (other reasons: n = 69). More details regarding the cohort have been published elsewhere [28]. We conducted a drop out analysis to compare characteristics of respondents with complete follow-up data and respondents who dropped out sometime after wave 3.

### Body mass index

During the interviews participants were asked to report their height and weight. Using the WHO thresholds, BMI categories were defined as follows: underweight (BMI < 18.5 kg/m^2^), normal weight (18.5 kg/m^2^ ≤ BMI < 25 kg/m^2^), overweight (25 kg/m^2^ ≤ BMI < 30 kg/m^2^), and obesity (BMI ≥ 30 kg/m^2^). For n = 84 (4.2 %) participants data on BMI was missing. These subjects were excluded from the analysis, resulting in a final sample size of n = 1,882 at wave 3 (wave 4: n = 1,575; wave 5: n = 1,219).

### Socio-demographic variables

Socio-demographic variables used for the analyses included: Sex, age groups (≤80 years, 81-83 years, 84-86 years, 87-89 years, ≥ 90 years), living situation (living alone in private household vs. others), educational status measured by CASMIN classification (elementary education, secondary education and tertiary education), family status (married vs. others) and having own children (no children versus ≥1 child). Furthermore, dummies for region were included in all regressions (results for region dummies are not shown in tables but are available upon request from the authors).

### Physical activity

Physical activity was assessed using a measure adapted from a scale developed by Verghese et al. [46]. Patients reported the frequency of physical activities as “every day”, “several times a week”, “once a week”, “less than once a week” and “never” for each activity in the last four weeks. To quantify physical activities, we considered cycling, gymnastics, swimming and long walks (dichotomized in each case: at least once a week vs. less than once a week/never). Though other activities (gardening, caring for grandchildren and other things) are available in the dataset, we focused on the four activities mentioned as we suppose that these cover a very wide range of physical activities.

### Other variables

Walking impairments were categorized according to the grade of mobility impairment (no impairment, aggravated walking and substantial mobility impairment/disability of walking). The Global Deterioration Scale (GDS) was used to assess cognitive impairment, ranging from 1 (no impairment) to 7 (severe impairment). In sensitivity analyses, we included data on depression (measured by the Geriatric Depression Scale ranging from 0 = no depression to 15 = severe depression) and comorbidity (presence/absence of 28 chronic conditions reported by the GP). If a comorbid condition was present, severity was rated by the GP on a 1 (mild) to 4 (severe) scale. A weighted count score was calculated as sum of severity ratings for conditions scored as present. Except for BMI mentioned above, the proportion of missing values was negligible.

### Statistical analyses

First, we provide descriptive statistics for individuals at baseline (wave 3) by BMI category. Second, by using cross-sectional regressions, we examined predictors of BMI (linear Ordinary Least Squares (OLS) regression) and overweight or obesity (logistic regression), respectively. Third, by exploiting the longitudinal data structure using panel econometric models, we investigated determinants of the above mentioned dependent variables. To detect gender effects, gender-specific regressions were additionally computed.

We used fixed effects (FE) regressions [7] to estimate effects of time dependent variables on BMI, and overweight or obesity, respectively. Hence, we assume that unobservable individual effects, such as genetic predisposition or personality, are correlated with the regressors x_it_. In such a case, a random effects regression is inconsistent since this model assumes that there is no correlation between unobservable individual effects and regressors (in detail: [7]). Because these unobservable individual effects were cancelled out by within-transformation in FE-regression (for more technical details: [7] and Additional file 1), time-constant unobserved heterogeneity is no longer a problem. This implies that we only exploit within-variation (therefore, the FE estimator is also called ‘Within-Estimator’). Thus, we can obtain causal estimates via FE-estimator (average treatment effect on the treated [4]), but generally internal validity should be interpreted with caution as we do not have a controlled stimulus. In order to deal with heteroskedasticity and autocorrelation, we computed cluster-robust standard errors [6, 44].

For logistic regression analyses, BMI was dichotomized into the categories overweight (1 if BMI ≥ 25) or otherwise (0). Analogously, BMI was dichotomized into the categories obesity (1 if BMI ≥ 30) or otherwise (0). This was done because we were interested in transitions from non-overweight to at least overweight and transitions from non-obesity to obesity.

In order to ensure the robustness of our findings in terms of significance, we tested various alternate model specifications. These models differ by adding (comorbidity, depression) or removing (cognitive impairment, mobility impairments) predictors that might be endogenous. The level of significance was set at α = .05, whereas exact p values were reported for p < .10. Statistical analysis was performed using Stata Release 13 (Stata Corp., College Station, Texas) on PC.

## Results

### Sample characteristics

Table 1 depicts descriptive findings. It is worth mentioning that we solely present variables included in our main model and time-invariant sociodemographic variables. Moreover, descriptive statistics over time are depicted in Table 2.

**Table 1:** Sample characteristics at wave 3, by BMI category

	Total	Underweight	Normal	Overweight	Obese	Cramer’s V and p-values^1^
	n (%)	n (%)	n (%)	n (%)	n (%)	
Total	1,882 (100)	35 (100)	837 (100)	752 (100.0)	258 (100)	
Age group						V = .105; p = .000
-80	297 (15,8)	1 (2,9)	92 (11)	144 (19,1)	60 (23,3)	
81-83	645 (34,3)	9 (25,7)	278 (33,2)	261 (34,7)	97 (37,6)	
84-86	533 (28,3)	12 (34,3)	257 (30,7)	210 (27,9)	54 (20,9)	
87-89	303 (16,1)	11 (31,4)	144 (17,2)	111 (14,8)	37 (14,3)	
90+	104 (5,5)	2 (5,7)	66 (7,9)	26 (3,5)	10 (3,9)	
Sex						V = .132; p = .000
Women	1,239 (65,8)	31 (88,6)	590 (70,5)	443 (58,9)	175 (67,8)	
Men	643 (34,2)	4 (11,4)	247 (29,5)	309 (41,1)	83 (32,2)	
Education						V = .070; p = .005
Elementary education	1,119 (59,5)	17 (48,6)	462 (55,2)	469 (62,4)	171 (66,3)	
Secondary education	539 (28,6)	14 (40)	265 (31,7)	193 (25,7)	67 (26)	
Tertiary education	224 (11,9)	4 (11,4)	110 (13,1)	90 (12)	20 (7,8)	
Living situation						V = .090; p = .002
Living alone in private household	951 (50,5)	18 (51,4)	457 (54,6)	339 (45,1)	137 (53,1)	
Others	931 (49,5)	17 (48,6)	380 (45,4)	413 (54,9)	121 (46,9)	
Family status						V = .120; p = .000
Married	656 (34,9)	10 (28,6)	249 (29,7)	314 (41,9)	83 (32,2)	
Others	1,224 (65,1)	25 (71,4)	588 (70,3)	436 (58,1)	175 (67,8)	
Own Children						V = .089; p = .002
No	381 (20,3)	14 (40)	188 (22,5)	134 (17,8)	45 (17,4)	
Yes	1,5 (79,7)	21 (60)	649 (77,5)	617 (82,2)	213 (82,6)	
Cycling						V = .079; p = .009
Never/<once a week	1,606 (85,8)	34 (97,1)	727 (87,5)	619 (82,9)	226 (87,6)	
At least once a week	265 (14,2)	1 (2,9)	104 (12,5)	128 (17,1)	32 (12,4)	
Gymnastics						V = .079; p = .009
Never/<once a week	1,077 (57,8)	21 (61,8)	443 (53,4)	458 (61,6)	155 (60,3)	
At least once a week	787 (42,2)	13 (38,2)	386 (46,6)	286 (38,4)	102 (39,7)	
Swimming						V = .025; p = .769
Never/<once a week	1,737 (93,2)	34 (97,1)	772 (93,3)	691 (92,8)	240 (93,4)	
At least once a week	127 (6,8)	1 (2,9)	55 (6,7)	54 (7,2)	17 (6,6)	
Long walk						V = .117; p = .000
Never / <once a week	866 (46,3)	21 (60)	367 (44,2)	324 (43,3)	154 (59,7)	
At least once a week	1,006 (53,7)	14 (40)	464 (55,8)	424 (56,7)	104 (40,3)	
Walking impairments						V = .115. p = .000
No walking impairments	916 (48,7)	9 (25,7)	442 (52,8)	369 (49,1)	96 (37,2)	
Aggravated Walking	749 (39,8)	14 (40)	298 (35,6)	319 (42,4)	118 (45,7)	
Substantial mobility impairment/disability of walking	217 (11,5)	12 (34,3)	97 (11,6)	64 (8,5)	44 (17,1)	
Global Deterioration Scale: Mean (SD)	2.0 (1.1)	2.9 (1.9)	2.0 (1.1)	1.9 (1.0)	1.8 (0.9)	p = .000
Comorbidities (weighted count): Mean (SD)	4.5 (3.9)	5.3 (4.8)	4.1 (3.5)	4.6 (3.7)	5.6 (5.0)	p = .000

^1^Analyses of variance were used for the metric variables (GDS and Comorbidities), and chi-squared tests (with Cramer’s V) for all other variables

Source: AgeCoDe (Wave 3), own calculations

**Table 2:** Descriptive statistics over time

	Wave 3	Wave 4	Wave 5
	n (%)	n (%)	n (%)
Total	1,882 (100)	1,575 (100)	1,219 (100)
Age group			
-80	297 (15,8)	3 (15,8)	0 (0,2)
81-83	645 (34,3)	530 (34,3)	167 (33,7)
84-86	533 (28,3)	509 (28,3)	470 (32,3)
87-89	303 (16,1)	360 (16,1)	346 (22,9)
90+	104 (5,5)	173 (5,5)	236 (11)
Sex			
Women	1,239 (65,8)	1053 (65,8)	813 (66,9)
Men	643 (34,2)	522 (34,2)	406 (33,1)
Education			
Elementary education	1,119 (59,5)	920 (59,5)	701 (57.5)
Secondary education	539 (28,6)	468 (28,6)	359 (29,5)
Tertiary education	224 (11,9)	187 (11,9)	159 (13,0)
Living situation			
Living alone in private household	951 (50,5)	792 (50,5)	593 (50,3)
Others	931 (49,5)	782 (49,5)	626 (49,7)
Family status			
Married	656 (34,9)	523 (34,9)	358 (33,2)
Others	1,224 (65,1)	1051 (65,1)	859 (66,8)
Own Children			
No	381 (20,3)	316 (20,3)	233 (19.1)
Yes	1,5 (79,7)	1258 (79,7)	985 (80.9)
Cycling			
Never / <once a week	1,606 (85,8)	1390 (85,8)	1097 (88,8)
At least once a week	265 (14,2)	175 (14,2)	116 (11,2)
Gymnastics			
Never/<once a week	1,077 (57,8)	937 (57,8)	744 (60)
At least once a week	787 (42,2)	624 (42,2)	464 (40)
Swimming			
Never/<once a week	1,737 (93,2)	1462 (93,2)	1148 (93,7)
At least once a week	127 (6,8)	98 (6,8)	63 (6,3)
Long walk			
Never/<once a week	866 (46,3)	773 (46,3)	707 (49,4)
At least once a week	1,006 (53,7)	792 (53,7)	507 (50,6)
Walking impairments			
No walking impairments	916 (48,7)	664 (48,7)	445 (42,2)
Aggravated Walking	749 (39,8)	712 (39,8)	580 (45,2)
Substantial mobility impairment/disability of walking	217 (11,5)	199 (11,5)	194 (12,6)
Global Deterioration Scale: Mean (SD)	2.0 (1.1)	2.1 (1.1)	2.1 (1.2)
Comorbidities (weighted count): Mean (SD)	4.5 (3.9)	4.6 (3.9)	3.9 (3.8)
BMI kg/m^2^: Mean (SD)	25.8 (4.2)	25.7 (4.3)	25.5 (3.9)

Of the n = 1,882 participants in wave 3, 65.8 % were female (Table 1). Mean age was 84.0 years (±3.3), ranging between 79 and 97 years^1^. The majority of participants had elementary education (59.5 %), lived alone in a private household (50.5 %), was not married (divorced, single and widowed: 65.1 %) and had own children (79.7 %). The proportion of participants who reported to perform cycling, gymnastics, swimming or long walks at least once a week were 14.2 %, 42.2 %, 6.8 %, 53.7 %, respectively. The mean weighted comorbidity score was 4.5 (±3.9) and the mean GDS score was 2.0 (±1.1).

### Cross-sectional analyses

#### Descriptive and bivariate analyses

Mean BMI was 25.8 kg/m^2^ (±4.2 kg/m^2^). Of all participants (women/men), 1.8 % were underweight (2.5 %/0.6 %), 44.5 % normal weight (47.6 %/38.4 %), 40.0 % overweight (35.8 %/48.1 %) and 13.7 % obese (14.1 %/12.9 %). Prevalence of obesity varied substantially by selected sample characteristics, with the highest prevalence rates found among participants who were aged ≤ 80, female, elementarily educated, living alone, not married, having own children, not taking long walks, and who had impaired mobility. Results were similar regarding highest prevalence rates of overweight. Bivariate statistical analyses indicated significant associations between all independent variables and BMI categories, except for swimming.

#### Multivariate analyses

##### BMI

BMI was positively associated with male gender. In the total sample and in both sexes, BMI was positively associated with younger age groups, lower educational level and impairments in walking, whereas cognitive impairment was negatively associated with BMI (Table 3). In men, performing gymnastics at least once a week was associated with a lower BMI, whereas in women, performing long walks at least once a week and having no own children was associated with a lower BMI.

**Table 3:** Cross-sectional regressions: predictors of BMI, overweight and obesity

	OLS-regressions (Beta-Coefficients reported)			Logistic regressions (Odd Ratios reported)					
Variables	BMI - all	BMI - women	BMI - men	Overweight - all	Overweight - women	Overweight - men	Obesity - all	Obesity - women	Obesity - men
81-83 years (Ref.: -80 years)	-1.075***	-0.834*	-1.418**	0.570***	0.534**	0.614*	0.636*	0.786	0.455*
	(0.299)	(0.400)	(0.464)	(0.422 - 0.770)	(0.362 - 0.789)	(0.379 - 0.995)	(0.440 - 0.920)	(0.489 - 1.264)	(0.245 - 0.845)
84-86 years	-1.651***	-1.677***	-1.675***	0.475***	0.399***	0.613+	0.371***	0.389***	0.333**
	(0.313)	(0.415)	(0.503)	(0.347 - 0.652)	(0.267 - 0.596)	(0.361 - 1.043)	(0.243 - 0.567)	(0.228 - 0.665)	(0.160 - 0.690)
87-89 years	-1.880***	-1.880***	-1.806**	0.452***	0.404***	0.533*	0.432***	0.515*	0.287**
	(0.342)	(0.447)	(0.550)	(0.317 - 0.644)	(0.258 - 0.633)	(0.294 - 0.969)	(0.268 - 0.697)	(0.286 - 0.930)	(0.116 - 0.709)
90+ years	-2.807***	-2.809***	-2.724***	0.260***	0.224***	0.295**	0.276***	0.245**	0.291+
	(0.478)	(0.615)	(0.783)	(0.157 - 0.432)	(0.119 - 0.423)	(0.119 - 0.727)	(0.129 - 0.591)	(0.0916 - 0.658)	(0.0817 - 1.039)
Sex (Ref.: Women)	0.568*			1.405**			1.041		
	(0.236)			(1.095 - 1.803)			(0.733 - 1.479)		
Secondary education (Ref.: Elementary education)	-0.652**	-0.736**	-0.398	0.745**	0.726*	0.780	0.784	0.786	0.767
	(0.225)	(0.281)	(0.358)	(0.598 - 0.927)	(0.559 - 0.943)	(0.511 - 1.190)	(0.571 - 1.076)	(0.538 - 1.148)	(0.418 - 1.409)
Tertiary education	-1.167***	-1.220*	-0.831*	0.581***	0.518*	0.677+	0.505**	0.491+	0.558
	(0.277)	(0.490)	(0.346)	(0.423 - 0.798)	(0.312 - 0.861)	(0.439 - 1.042)	(0.301 - 0.847)	(0.214 - 1.125)	(0.278 - 1.123)
Living alone in private household (Ref.: No)	-0.199	-0.0491	-0.597	0.849	0.828	0.935	0.942	1.264	0.479+
	(0.263)	(0.323)	(0.450)	(0.644 - 1.121)	(0.598 - 1.147)	(0.532 - 1.643)	(0.637 - 1.393)	(0.783 - 2.039)	(0.213 - 1.076)
Married (Ref.: Others)	-0.242	-0.0623	-0.588	1.016	1.027	1.031	0.775	1.049	0.462*
	(0.304)	(0.452)	(0.404)	(0.745 - 1.387)	(0.677 - 1.560)	(0.628 - 1.695)	(0.499 - 1.204)	(0.579 - 1.900)	(0.235 - 0.910)
Own Children (Ref.: No)	0.792***	0.938***	0.251	1.288*	1.245	1.356	1.291	1.366	1.002
	(0.227)	(0.272)	(0.408)	(1.014 - 1.638)	(0.937 - 1.654)	(0.844 - 2.177)	(0.901 - 1.850)	(0.893 - 2.089)	(0.494 - 2.031)
Cycling, at least once a week (Ref.: Never / <once a week)	-0.182	-0.439	0.0906	1.231	1.108	1.363	0.851	0.683	0.969
	(0.242)	(0.388)	(0.295)	(0.915 - 1.657)	(0.723 - 1.700)	(0.889 - 2.089)	(0.550 - 1.317)	(0.352 - 1.323)	(0.521 - 1.804)
Gymnastics, at least once a week	-0.393*	-0.159	-0.955***	0.723**	0.823	0.550**	0.942	0.984	0.835
	(0.196)	(0.257)	(0.287)	(0.592 - 0.884)	(0.644 - 1.051)	(0.385 - 0.787)	(0.707 - 1.254)	(0.698 - 1.387)	(0.486 - 1.434)
Swimming, at least once a week	0.220	0.302	0.0571	1.089	0.994	1.173	1.098	1.225	0.866
	(0.437)	(0.696)	(0.381)	(0.740 - 1.602)	(0.600 - 1.647)	(0.631 - 2.182)	(0.630 - 1.913)	(0.608 - 2.466)	(0.338 - 2.216)
Long Walk, at least once a week	-0.722***	-0.837**	-0.478	0.910	0.939	0.859	0.542***	0.524**	0.540*
	(0.208)	(0.278)	(0.320)	(0.733 - 1.130)	(0.716 - 1.230)	(0.589 - 1.252)	(0.396 - 0.741)	(0.355 - 0.773)	(0.310 - 0.942)
Aggravated walking (Ref.: no impairment)	0.754***	0.618*	0.962*	1.676***	1.558**	1.861**	1.547**	1.585*	1.475
	(0.219)	(0.275)	(0.383)	(1.344 - 2.090)	(1.191 - 2.037)	(1.240 - 2.793)	(1.124 - 2.131)	(1.076 - 2.334)	(0.814 - 2.675)
Substantial mobility impairment/disability of walking	0.939*	0.989*	0.832	1.577*	1.713*	1.311	2.701***	2.608**	2.911*
	(0.389)	(0.487)	(0.643)	(1.094 - 2.273)	(1.100 - 2.668)	(0.674 - 2.552)	(1.670 - 4.370)	(1.444 - 4.712)	(1.242 - 6.823)
Global Deterioration Scale	-0.602***	-0.656***	-0.454**	0.791***	0.771***	0.829+	0.745***	0.780**	0.676**
	(0.0972)	(0.121)	(0.166)	(0.716 - 0.874)	(0.683 - 0.869)	(0.687 - 1.000)	(0.640 - 0.866)	(0.652 - 0.935)	(0.505 - 0.905)
Constant	28.51***	28.79***	28.80***	3.479***	4.798***	2.883*	0.547	0.407+	1.059
	(0.518)	(0.672)	(0.832)	(2.009 - 6.024)	(2.441 - 9.431)	(1.091 - 7.614)	(0.261 - 1.146)	(0.164 - 1.008)	(0.270 - 4.157)
Observations	1,858	1,230	628	1,858	1,230	628	1,858	1,230	628
(Pseudo-)R^2^	0.089	0.098	0.094	0.061	0.065	0.053	0.061	0.068	0.090

Comments: Ordinary Least Squares (OLS) Regressions: Cluster-robust standard errors in parentheses; Logistic Regressions: 95 % CI in parentheses

***p < 0.001, ** p < 0.01, * p < 0.05, + p < 0.10; Region dummies were included in all regressions

Source: AgeCoDe (Wave 3), own calculations

##### Overweight/obesity

Male gender was positively associated with overweight. In the total sample and in both sexes, overweight and obesity were positively associated with younger age groups and walking impairments, and negatively associated with cognitive impairment and long walks (obesity only). Furthermore, in women and in the total sample, overweight was positively associated with lower educational level. In the total sample, overweight was positively associated with own children. In men, overweight was negatively associated with performing gymnastics.

### Longitudinal analyses

#### BMI

In the total sample and in both sexes, BMI significantly decreased with increasing age (Table 4). Interestingly, starting gymnastics at least once a week increased BMI in women by 0.29 units, whereas BMI in men decreased by -0.40 units (p = .09). Furthermore, increasing impairments in walking (e. g. substantial mobility impairment/disability of walking in the total sample: β = -0.97) and increasing cognitive impairment decreased BMI in women and in the total sample. For example, when the Global Deterioration Scale in the total sample increased by one unit, the BMI decreased by -0.16 units.

**Table 4:** Longitudinal regressions (Within-Estimations): predictors of BMI, overweight and obesity

	FE-regressions (Beta-Coefficients reported)			Conditional logistic FE-regressions (Odd Ratios reported)					
Variables	BMI - all	BMI - women	BMI - men	Overweight - all	Overweight - women	Overweight - men	Obesity - all	Obesity - women	Obesity - men
81-83 years (Ref.: -80 years)	-0.427*	-0.218	-0.699*	0.500+	0.256*	0.635	0.462+	0.545	0.468
	(0.173)	(0.180)	(0.311)	(0.243 - 1.032)	(0.0703 - 0.928)	(0.245 - 1.645)	(0.201 - 1.065)	(0.151 - 1.971)	(0.152 - 1.446)
84-86 years	-0.767***	-0.649**	-0.910**	0.276**	0.135**	0.376+	0.245**	0.219*	0.386
	(0.200)	(0.239)	(0.315)	(0.119 - 0.638)	(0.0332 - 0.545)	(0.117 - 1.206)	(0.0924 - 0.650)	(0.0521 - 0.917)	(0.0876 - 1.700)
87-89 years	-1.108***	-1.030***	-1.073**	0.185***	0.0938**	0.199*	0.162**	0.158*	0.211
	(0.236)	(0.298)	(0.339)	(0.0693 - 0.492)	(0.0207 - 0.426)	(0.0397 - 0.996)	(0.0467 - 0.565)	(0.0277 - 0.898)	(0.0222 - 1.998)
90+ years	-0.910**	-0.789+	-0.998*	0.175**	0.100*	0.117*	0.313	0.254	0.938
	(0.312)	(0.407)	(0.407)	(0.0511 - 0.601)	(0.0169 - 0.592)	(0.0140 - 0.978)	(0.0661 - 1.482)	(0.0321 - 2.002)	(0.0524 - 16.77)
Living alone in private household (Ref.: No)	-0.0712	0.00237	-0.425+	1.514	1.223	3.257	1.106	3.561	0.0401**
	(0.165)	(0.211)	(0.245)	(0.773 - 2.963)	(0.582 - 2.566)	(0.651 - 16.29)	(0.331 - 3.694)	(0.672 - 18.89)	(0.00403 - 0.399)
Married (Ref.: Others)	-0.0890	-0.0387	-0.292	0.686	0.797	0.419	1.414	5.371+	0.383
	(0.342)	(0.607)	(0.340)	(0.247 - 1.907)	(0.139 - 4.576)	(0.0711 - 2.464)	(0.378 - 5.294)	(0.728 - 39.64)	(0.0613 - 2.391)
Cycling, at least once a week (Ref.: Never/<once a week)	-0.279	-0.335	-0.239	0.673	0.719	0.715	1.452	1.993	1.998
	(0.181)	(0.314)	(0.212)	(0.323 - 1.402)	(0.275 - 1.884)	(0.228 - 2.247)	(0.423 - 4.980)	(0.209 - 19.03)	(0.490 - 8.155)
Gymnastics, at least once a week	0.0729	0.293*	-0.404+	1.092	1.392	0.521	1.189	1.148	1.407
	(0.123)	(0.140)	(0.238)	(0.696 - 1.715)	(0.800 - 2.421)	(0.218 - 1.247)	(0.671 - 2.109)	(0.574 - 2.297)	(0.501 - 3.948)
Swimming, at least once a week	0.286	0.419	0.0254	0.822	0.652	1.501	1.123	2.152	0.926
	(0.404)	(0.590)	(0.259)	(0.360 - 1.879)	(0.227 - 1.875)	(0.372 - 6.059)	(0.314 - 4.011)	(0.147 - 31.53)	(0.149 - 5.747)
Long Walk, at least once a week	0.0358	-0.0558	0.181	1.282	1.216	1.366	0.745	0.632	1.272
	(0.116)	(0.144)	(0.193)	(0.851 - 1.931)	(0.705 - 2.097)	(0.686 - 2.721)	(0.420 - 1.321)	(0.332 - 1.202)	(0.404 - 4.003)
Aggravated walking (Ref.: no impairment)	-0.215	-0.378*	0.0991	0.815	0.886	0.528	0.959	0.846	1.450
	(0.136)	(0.187)	(0.148)	(0.477 - 1.393)	(0.452 - 1.737)	(0.177 - 1.576)	(0.482 - 1.908)	(0.358 - 1.998)	(0.484 - 4.343)
Substantial mobility impairment/disability of walking	-0.969***	-1.176***	-0.539+	0.409*	0.259*	0.660	0.337	0.533	0.200
	(0.214)	(0.272)	(0.322)	(0.188 - 0.888)	(0.0877 - 0.767)	(0.180 - 2.426)	(0.0908 - 1.253)	(0.103 - 2.760)	(0.0221 - 1.813)
Global Deterioration Scale	-0.160*	-0.202*	-0.0733	1.015	1.048	0.877	0.754	0.689	0.737
	(0.0641)	(0.0854)	(0.0927)	(0.762 - 1.354)	(0.741 - 1.483)	(0.520 - 1.482)	(0.536 - 1.059)	(0.418 - 1.134)	(0.407 - 1.333)
Constant	26.98***	26.65***	27.50***						
	(0.295)	(0.348)	(0.442)						
Observations	4,620	3,072	1,548	780	501	279	470	287	183
(Pseudo)R^2^	0.030	0.036	0.046	0.060	0.086	0.084	0.088	0.124	0.152
Number of Individuals	1,925	1,277	648	282	182	100	169	104	65
Sargan-Hansen Test (FE)/Hausman (CLFE): p-value	.000	.000	.000	.000	.000	.010	.000	.049	.055

Ordinary Least Squares (OLS) Regressions: Cluster-robust standard errors in parentheses; Logistic Regressions: 95 % CI in parentheses

*** p < 0.001, ** p < 0.01, * p < 0.05, + p < 0.10; Region dummies were included in all regressions

Source: AgeCoDe (Wave 3-5), own calculations

#### Overweight and obesity

The risk (odds) of becoming overweight or obese decreased with increasing age. However, for obesity this was only true in women. Interestingly, the risk of obesity in men strongly decreased when men changed to living alone [OR: .04 (.00-.40)]. Moreover, the occurrence of substantial mobility impairments or walking disabilities decreased the risk for overweight in women [OR: .26 (.09-.77)] and in the total sample [OR: .41 (.19-.89)].

### Alternate model specifications

Robustness was checked by comparing the baseline models (Table 3 and Table 4) with alternate models (results not shown, but are available upon request from authors). In a first and a second alternate specification, we additionally controlled for comorbidity (model I) and depression (model II), respectively. In a third and fourth specification, we excluded cognitive impairment (model III) and mobility impairments (model IV), respectively. In summary, it can be stated that these specifications underlined the robustness of our baseline models (in terms of significant predictors).

## Discussion

### Main findings

More than half of the sample was overweight or obese with the prevalence of excess weight differing by various sample characteristics. Highest prevalence rates of both overweight and obesity were found among participants aged ≤ 80 years (79 or 80 years), severely impaired in mobility and not taking long walks at least once a week.

Cross-sectional regressions showed that BMI was positively associated with younger age, severe walking impairments and negatively associated with cognitive impairments. Overweight and obesity were positively associated with younger age groups, elementary education, walking impairments and physical inactivity, whereas cognitive impairment was negatively associated with overweight and obesity. Besides, male gender was positively associated with overweight.

Longitudinal regressions, as opposed to cross-sectional regressions, showed that predominantly severely impaired walking abilities, besides age, drastically lowered BMI. Moreover, cognitive impairment reduced BMI in women. With older age brackets the probability of transitions to overweight as well as obesity decreased noticeably. Again, contrary to cross-sectional results, occurrence of severe walking impairments reduced the risk of changes to overweight considerably.

### Comparison to previous studies

Since it is difficult to compare findings of self-reported BMI (srBMI) and measured (mBMI), we explicitly state the method of measurement used in previous studies in parentheses (srBMI, mBMI or, more generally, systematic review).

#### Cross-sectional findings

This study adds to data on the prevalence of excess weight in the German adult population, as available studies were restricted to samples aged < 80 years [31] (mBMI). Prevalence of excess weight in subjects aged ≥ 80 years is lower compared to younger adults and similar to subjects aged 30-39 years [31]. This comparatively low prevalence may be partly explained by a reduction in energy intake and unintentional weight loss, which is frequent in older adults. This may suggest underlying diseases as well as impending death [21] (systematic review). Yet, in the total sample, prevalence rates of obesity found in our study were considerably lower compared to other European studies, in particular from Spain [20] (mBMI) and Italy [36] (mBMI). The Spanish study conducted in 2001 reported a high prevalence in individuals aged ≥ 80 (M: 19.4 %; F: 29.2 %). In 1992/1993, prevalence of obesity among Italian men and women aged 80-84 years was 7.7 % and 25.4 %, respectively.

Association of BMI/excess weight with socio-demographic variables found in our study are mostly in line with previous literature: BMI significantly decreased with age [29] (mBMI) and higher education [10] (systematic review), while risk for excess weight was higher for younger and less educated [35] (mBMI). Findings regarding sex - higher BMI and excess weight in men - are in line with the literature [24] (srBMI). However, in the literature, effects of gender were not consistently found and varied in direction [20].

Comparable to Weng et al. [48] (srBMI), a significant association between having children and overweight, but not for obesity, was detected. When we used number of children instead of a dummy-variable like Weng et al. [48], we also obtained significant associations with obesity, with marked sex differences.

Also in line with the literature [47] (systematic review), the strong association between BMI and walking impairments can simply be explained by low energy expenditure as a consequence of difficulties in moving around, contributing to a higher BMI.

The strong association between cognitive impairment and BMI has also been reported in the literature [2] (srBMI) and can be explained by frailty [40] of severely impaired individuals (GDS > 5) who had mean BMI of 22.1 (±3.9). However, the relation between cognitive impairment and BMI is complex and literature is inconclusive [14, 19, 22, 25, 26] (mBMI) and [43] (systematic review).

#### Longitudinal findings

##### BMI

Our longitudinal findings revealed a negative effect of old age on BMI. This supports previous findings for Austria [37] (mBMI) and Canada [42] (mBMI) and can be partly explained by the fact that with old age even lean body mass decreases (age-related sarcopenia) [18] (mBMI).

Physical activities raised BMI. Most probably, this finding is caused by an increase in muscle mass of thin women. This is supported by FE-regressions restricted to BMI < 23 kg/m^2^ (first quartile), in which physical activities raised BMI significantly (total sample: p < .01; women: p < .001), whereas if we restricted the FE-regression to other BMI values (e. g. BMI > 25 kg/m^2^, BMI > 28 kg/m^2^), no significant effects were observed.

##### Overweight and obesity

Corresponding to these results, starting physical activities did not increase the risk of becoming overweight and obese, respectively. Findings regarding living situation for men should be interpreted with caution since this regressor varies little over time.

Contrary to cross-sectional findings, when severe walking impairments occurred, risk of changes to overweight was reduced substantially. This contrast might be explained by frailty which is highly associated with walking impairments. More specifically, mobility impairment might be part of a useful screen for frailty [41].

### Strengths and limitations

A key strength is that we draw on an almost representative sample of older adults in Germany since subjects were recruited via GP offices and nearly everyone of this age bracket has GP visits. Furthermore, we add to the scarce literature on overweight and obesity in old age. Moreover, we have shown that the use of FE estimators is decisive to obtain unbiased estimates in BMI regressions (results not shown, but are available upon request from authors). This is caused by the control of unobserved heterogeneity.

We cannot rule out the possibility that there might be a simultaneity bias between mobility impairments and BMI. Theoretically, simultaneity bias can be solved by Panel-IV-estimator. Empirically, we decided against IV-technique since the problem of weak instruments was present.

Due to data availability, we were restricted to BMI as a measure of excess weight indicating need for weight management. Hence, alternative and important measures [38], such as waist-to-height ratio, waist-to-hip ratio or waist circumference cannot be used to validate our findings across instruments. Thus, it might be possible that our measure of weight underestimates obesity because old age individuals tend to have lower lean body mass [15]. However, height loss in older individuals influences BMI and masks weight loss partially.

Likewise, for reasons of data availability, we could not include shifts in eating habits. Moreover, we could not distinguish between intended and unintended weight loss due to data restrictions [34]. Since only self-reported data were available regarding height and weight, it is most likely that BMI is biased downwards as individuals tend to underestimate weight and overestimate height [11].

Furthermore, panel attrition caused by time-dependent variables might have biased our FE-estimates. We tested whether there were significant differences at wave 3 between individuals with complete follow-up data and individuals who dropped out after wave 3, regardless of the exact time. This latter group was initially older, physically less active, more depressed and cognitively impaired (results not shown, but are available upon request from authors). Thus, the representativeness is limited due to attrition bias.

## Conclusions

Substantial differences between regressors (e. g. mobility impairment, physical activities) in cross-sectional and longitudinal setting exist, emphasizing the complex nature of excess weight in old age. More research is needed to disentangle these effects. Additionally, since controlling for unobserved heterogeneity is crucial, more longitudinal studies are required. Moreover, due to the fact that most of the previous findings concerning BMI and excess weight in the elderly were based on individuals aged 60+ or 65+ years [17, 49], further research is required regarding individuals in very old age.

Furthermore, it is unclear whether a reduction in BMI in old age is generally desirable, taking into account the “obesity paradox”. Many other factors need to be taken into account such as body fat percentage, medical history or frailty. This, in turn, raises also the question whether the WHO cut-off point of 25 kg/m^2^ for overweight in old age is too restrictive in this age-bracket [8, 12, 13, 23].

## Additional file

Additional file 1:
**Fixed effects regression (technical details).**

## Footnotes

1 In wave 4, mean age was 85.4 years (±3.2, min: 80, max: 98), while in wave 5 mean age was 86.8 years (±3.0, min: 81, max: 98).
