# Supplementary material for: Prevalence and determinants of overweight and obesity in old age in Germany
Source: BMC Geriatr. 2015 Jul 14;15:83. doi: 10.1186/s12877-015-0081-5 (PMC4499904; doi:10.1186/s12877-015-0081-5)
Supplement: Additional file 1: — Fixed effects regression (technical details). [file 12877_2015_81_MOESM1_ESM.docx]

**Additional file 1 – Fixed effects regression (technical details)**

The error-components model is our starting point:

$$y_{it}=x_{it}^{'}\beta+\alpha_{i}+\varepsilon_{it}$$

By averaging this equation over time for each individual i (between-transformation), we get:

$$\bar{y}_{i}=\bar{x}_{i}^{'}\beta+\alpha_{i}+\bar{\varepsilon}_{i}$$

By subtracting the second equation from the first equation for each t (within-transformation), we get:

$${y_{it}-\bar{y}}_{i}=\left( x_{it}-\bar{x}_{i} \right)'\beta+\left( \varepsilon_{it}-\bar{\varepsilon}_{i} \right)$$

Now, the idiosyncratic error-term α_i_ is removed. We have “time-demeaned” our data (the between variation was subtracted). This model can be estimated by OLS.
